# Circulating endothelial progenitor cells during pregnancy in multiple sclerosis

**DOI:** 10.1007/s10072-020-04648-3

**Published:** 2020-08-17

**Authors:** Giulia Mallucci, Fausta Beneventi, Roberto Bergamaschi, Cristina Bizzotto, Chiara Cavagnoli, Irene De Maggio, Camilla Bellingeri, Cristina Monti, Gianluca Viarengo, Arsenio Spinillo

**Affiliations:** 1 Department of Brain and Behavioral Sciences, University of Pavia, Pavia, Italy; 2 Multiple Sclerosis Center, IRCCS Mondino Foundation, Via Mondino 2, 27100 Pavia, Italy; 3 Department of Obstetrics and Gynaecology, IRCCS Foundation Policlinico San Matteo and University of Pavia, Pavia, Italy; 4 Department of Public Health Experimental and Forensic Medicine, Unit of Biostatistics and Clinical Epidemiology, University of Pavia, Pavia, Italy; 5 Immunohaematology and Transfusion Service, IRCCS Foundation Policlinico San Matteo, Pavia, Italy

**Keywords:** Multiple sclerosis, Pregnancy, Circulating endothelial progenitor cells, EPC, Cord blood

## Abstract

**Background:**

Endothelial progenitor cells (EPCs) have been shown to increase during physiological pregnancy and are believed to play a fundamental role in the process of placentation. Reduced levels of EPCs during pregnancy have been associated with preeclampsia and miscarriage. Women with multiple sclerosis (MS) are not at increased risk of preeclampsia nor of general adverse obstetric outcome, in contrast with some other autoimmune diseases.

**Objective:**

The aim of this study was to evaluate circulating EPCs levels in pregnant patients with MS.

**Methods:**

CD34+ and CD133+ were longitudinally detected by flow cytometry in the maternal plasma of 29 healthy controls and 9 MS patients and in the cord blood of their newborns.

**Results:**

EPCs were affected by pregnancy with the same trend in both groups (CD34+ *p* = 0.0342; CD133+ *p* = 0.0347). EPCs during pregnancy were increased in MS (mean ± SD: CD34+ cells 0.038 ± 0.010; CD133+ 0.024 ± 0.009) with respect to healthy controls (mean ± SD: CD34+ cells 0.022 ± 0.006; CD133+ 0.016 ± 0.004), CD34+ *p* = 0.0004; CD133+ *p* = 0.0109. EPCs levels of the cord blood of MS patients' newborns mild correlated with maternal EPC levels at delivery (CD34+: spearman’s Rho 0.658, *p* = 0.054; CD133+: spearman’s Rho 0.758, *p* = 0.018).

**Conclusions:**

This work identified increased circulating EPC levels during pregnancy, following the same trend both in MS patients and healthy controls. Despite the similar trend, the levels of circulating EPCs were significantly higher in MS patients with respect to the control population. A correlation was also found in MS patients between cord blood EPCs and circulating EPCs at delivery.

**Electronic supplementary material:**

The online version of this article (10.1007/s10072-020-04648-3) contains supplementary material, which is available to authorized users.

## Introduction

Multiple sclerosis (MS) is a chronic inflammatory and degenerative demyelinating disease of the central nervous system of presumed autoimmune aetiology [1, 2].

The disease mainly affects young women of childbearing age, and more recent data show that more than 50% of childbearing age MS women get pregnant after diagnosis [3].

Pregnancy has a protective effect on MS course, progressively decreasing the risk of relapses up to 70% by the end of the third trimester due to multiple factors, which are likely involved [4]. However, the current literature suggests that pregnancy has no effect on long-term outcomes of MS in terms of disability [5]. Vice versa, MS does not seem to affect the course of pregnancy: the risks of complications during pregnancy and of adverse pregnancy outcomes are not significantly different compared with healthy controls [6, 7].

Circulating endothelial progenitor cells (EPCs), expressing CD34 and CD133, are believed to play a significant role both in vasculogenesis and vascular repair. Since their identification by Asahara et al. in 1997 [8], most studies on EPCs focused on their role in cardiovascular health and on their potential therapeutic applications [9].

During uncomplicated pregnancy, the number of circulating EPCs seems to increase, possibly as a result of neo-angiogenesis that occurs during placentation [10–12]. On the other hand, a decreased number of circulating EPCs have been associated with preeclampsia, a disease notably characterized by defective placental vascularization [10, 13] and with increased risk of miscarriage [14, 15].

Endothelial dysfunction is a known feature of some autoimmune connective tissue diseases; altered levels and function of EPCs have been detected in rheumatoid arthritis (RA), systemic sclerosis (SSc) and systemic lupus erythematosus (SLE) [16–21]. Interestingly, these conditions are associated with increased risk of adverse obstetric outcome [22–24].

MS shares several features with RA, SSc and SLE, notably the autoimmune component. However, unlike other autoimmune diseases, MS patients are not at increased risk of general adverse obstetric outcome nor of foetal or neonatal complications [6, 7]. The assessment of circulating EPC levels during pregnancy in MS patients may shed some light on the difference observed in pregnancy outcome favourability in MS patients relative to that observed in patients diagnosed with other autoimmune diseases.

## Materials and methods

This non-interventional prospective study was approved by the medical ethics committee of IRCCS Policlinico San Matteo of Pavia (Current Research Project N. 10,901-rcr2017i-23 years 2017–2020) and was conducted according to the principles of the Declaration of Helsinki.

The study was run in collaboration between the Department of Obstetrics and Gynecology, IRCCS Policlinico San Matteo Pavia, and the MS Centre, IRCCS Mondino Foundation of Pavia.

### Patients and controls

A total of 38 subjects, 9 relapsing remitting MS patients (MS) and 29 healthy controls (CTRL), were enrolled in the study at 11–13 weeks of gestation from April 2017 to April 2019. All recruited subjects signed an informed consent form. MS patients had diagnosis according to McDonald Criteria [25]. Three controls for each MS case were selected from obstetrics, frequency-matched by age. Healthy controls and MS patients were prospectively followed up until delivery. Neither multiple pregnancies nor pregnancies with chromosomal or foetal abnormalities were observed in these 38 subjects.

### Neurological outcome

According to clinical practice, a neurological exam was performed during the first trimester (i.e. 11–13 weeks of gestation) and the third trimester (i.e. 28–32 weeks of gestation). During the first trimester consult, medical history, MS history, demographic and clinical data were also recorded. At each examination, Expanded Disability Status Scale (EDSS) [26], MS relapses, MS radiological activity and adverse events (AE) were reported. None of the MS patients was on treatment for MS (disease modifying treatment, DMT) during pregnancy or lactation. MS relapse was defined as an episode of neurological symptoms that lasts at least 24 h in the absence of fever and infection. MS radiological activity was defined as contrast-enhanced lesions or the appearance of new T2-hyperintense lesions, compared with the previous scan. Chronic disability progression (CDP) was defined as (i) ≥ 1.5-point increase if EDSS = 0 at baseline, or (ii) ≥ 1.0-point increase if EDSS = 0.5–4.5 at baseline, or (iii) ≥ 0.5-point increase if EDSS ≥ 5.0 at baseline, confirmed at 3 months. NEDA-3 (no evidence of disease activity) status was reported [27]. In detail, the three assessed components were (i) no CDP, (ii) no relapse activity, and (iii) no radiological activity.

### Obstetric outcome

After the first trimester (enrolment), MS patients and CTRL were followed up monthly with obstetric clinical and ultrasonographic evaluations.

Foetal growth restriction (FGR) was diagnosed when the abdominal foetal circumference at ultrasonographic examination fell below the 10th percentile of our local reference curves [28], confirmed on at least two consecutive measurements taken 2 weeks apart after the standard US obtained at 18–22 weeks of pregnancy, and pulsatility index (PI) of umbilical artery was higher than the 95th percentile of reference curves signalling a reduced perfusion of the fetoplacental unit [29].

Pre-eclampsia was diagnosed according to standard criteria. Stored maternal blood samples from first (11–13 weeks) and third (28–32 weeks) trimesters and delivery and stored cord blood were used to evaluate EPC count.

The mean uterine artery PI was evaluated in the first and third trimesters according to standard methods. PI of uterine arteries was considered abnormal when the values were higher than the 95th percentile of reference curves.

### Blood samples processing

Blood was collected from different participants at 11–13 weeks (first trimester), at 28–32 weeks (third trimester), at delivery and from cord blood and was used to evaluate EPC count.

Blood samples were freshly analysed by lyse-and-wash whole blood staining procedure. Surface staining was performed on ice for 20 min, and the cells were then analysed on a two-laser, six-colour FACSCanto flow cytometer. Multiplexed dilutions of monoclonal antibodies (mAbs) were used to characterize lymphocyte populations.

### Flow cytometry

The populations of CD34+ and CD133+ endothelial progenitor cells were measured by flow cytometry utilizing a Beckman Coulter Navios (Indianapolis, IN, USA) and were expressed as percent cell count/μL. The following antibodies were used: anti CD45 FITC and anti CD34 PE by Beckman Coulter (Indianapolis IN, USA), anti CD133 APC by Miltenyi Biotec (Bergisch Gladbach, Germany). The software utilized for acquisitions and analyses was Beckman Coulter Navios Software (Indianapolis IND, USA).

The positive population for both CD34 and CD133 antigens has been defined by a Boolean strategy utilizing three dot plots: CD45 vs side scatter to define leucocytes, CD34 vs side scatter to reveal CD34 positive cells and CD133 vs side scatter for CD133 positive cells.

### Statistical analyses

The feature description was summarized by the appropriate descriptive statistics. The comparison between MS and CTRL demographical and clinical characteristics was evaluated by Mann-Whitney *U* test or unpaired Student *t* test, as appropriate, for quantitative variables; chi-square or Fisher exact tests were applied to explore categorical variables’ differential distribution.

To study the variation of CD34+ and CD133+ cells’ endpoint variables during pregnancy, a non-parametric approach was chosen, composed by three steps. (1) Friedman test was used for the evaluation of the effect of gestational age (repeated time points for each woman: 11–13, 28–32 gestational weeks and time of delivery) independently from the effect of clinical status, followed, when significant, by post hoc sign tests with correction for multiple comparisons. (2) Mann-Whitney *U* tests were carried out, for the evaluation of the effect of clinical status, independently from the gestational age effect. Specifically, we compared the global median value of CD34+ and CD133+ cells between MS and CTRL, followed by the post hoc comparisons at the different gestational time points. (3) The statistical interaction between gestational age and clinical status (MS versus CTRL) was evaluated by graphical techniques.

To study the variation of CD34+ and CD133+ cells’ endpoint variables in the cord blood, we used Mann-Whitney *U* test.

Spearman’s rank correlation was used to identify a correlation between CD34+ and CD 133+ cells at delivery and in the cord blood.

A *p* value below 0.05 was considered statistically significant. All the analyses were performed using the STATA 15 statistical software (Stata Corporation, College Station, TX, USA).

## Results

### Demographic and clinical characteristics of MS patients and CTRL

All 38 participants completed the study. There were no dropouts, and none of the included participants was excluded from the analysis.

Demographic and clinical characteristics of individuals are summarized in Table 1. Age and parity were not significantly different between MS and CTRL, as well as gestational age, BMI, smoking/alcohol habits and rates of pregnancy complications. The frequency of previous miscarriages was increased in MS compared with CTRL, but within the healthy range [30]. Birth weight was higher in MS patients compared with CTRL but within the physiological range.

**Table 1:** Obstetrics outcomes of the study sample by clinical status. BMI = body mass index; CTRL = healthy control; MS = multiple Sclerosis; SGA = small for gestational age; NICU = neonatal intensive care unit; SD = standard deviation. Tests applied: *Mann-Whitney *U* test; °Pearson’s test; ^§^Fisher exact test

	MS	CTRL	*p* value
	*(n = 9)*	*(n = 28)*	
Age, years, mean (SD)	32.33 (4.90)	32.38 (4.81)	1.000*
Nulliparous	5 (55,5)	8 (28.57)	0.122°
Previous miscarriages, *n* (%)	3 (33,3)	1 (3.57)	0.035^*§*^
Smoking during pregnancy, *n* (%)	0	1 (3.57)	1.000^*§*^
BMI at delivery, Kg/m2, mean (SD)	24.63 (3.40)	21.80 (2.14)	0.210*
Weeks at delivery, mean (SD)	39.966 (1.39)	39.63 (0.84)	0.447*
Labour induction, *n* (%)	2 (22.2)	4 (14.28)	0.441^*§*^
Caesarean section, *n* (%)	3 (33)	6 (21.43)	0.739^*§*^
Preeclampsia, *n* (%)	1 (11.1)	1 (3.57)	0.442^*§*^
Fetal growth restriction, *n* (%)	0	0	–
SGA, *n* (%)	1 (11.1)	1 (3.57)	0.422^*§*^
NICU admission, *n* (%)	0	0	–
Neonatal weight, g, mean (SD)	3602.22 (501.84)	3331.90 (230.46)	0.031*

**Fig. 1:**
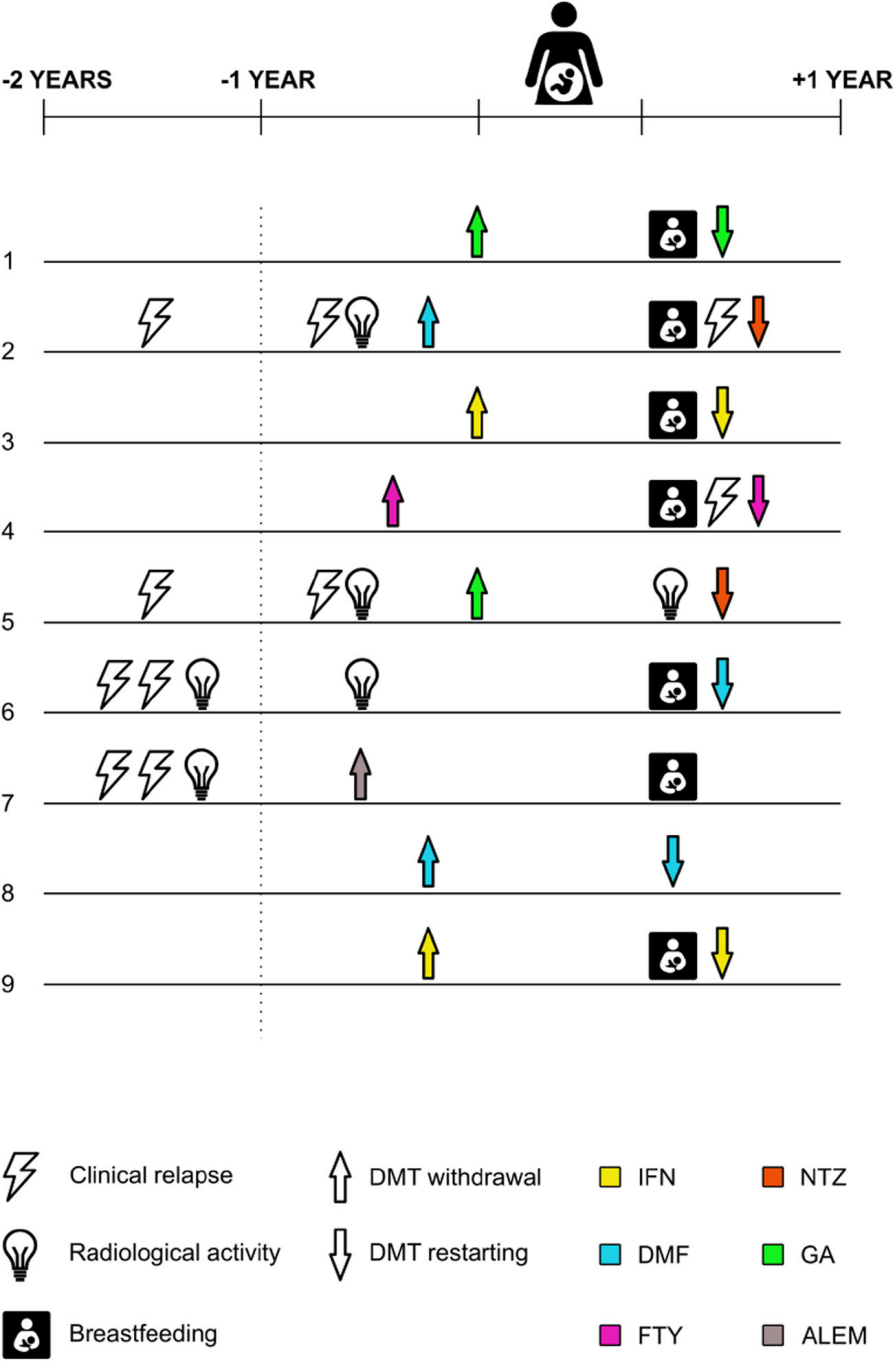
Clinical and radiological characteristics of included pregnant women with multiple sclerosis. DMT = disease modifying treatment, IFN = interferon beta 1b; DMF = dimethyl fumarate; FTY = fingolimod; NTZ = natalizumab, GA = glatiramer acetate; ALEM = alemtuzumab

Among MS patients, 6 out of 9 (67%) were NEDA 1 year before the onset of pregnancy, and 5 (55%) were NEDA 2 years before the onset of pregnancy. Mean MS duration at pregnancy was 8.33 ± 4.33 years. Median EDSS during the first trimester was 1.0 (min-max: 0–3.5) and during third trimester was 1.0 (min-max: 0–3.5). None of MS patients had a clinical relapse during pregnancy, but disease activity within 3 months from delivery was registered in 1/3 of patients. EDSS 3 months postpartum was 1.0 (min-max: 0–4.0). Clinical characteristics of individuals are summarized in Fig. 1**.**

### CD34 and CD133 population

A total of 114 observations (three observations per individual) were available for analysis.

**Fig. 2:**
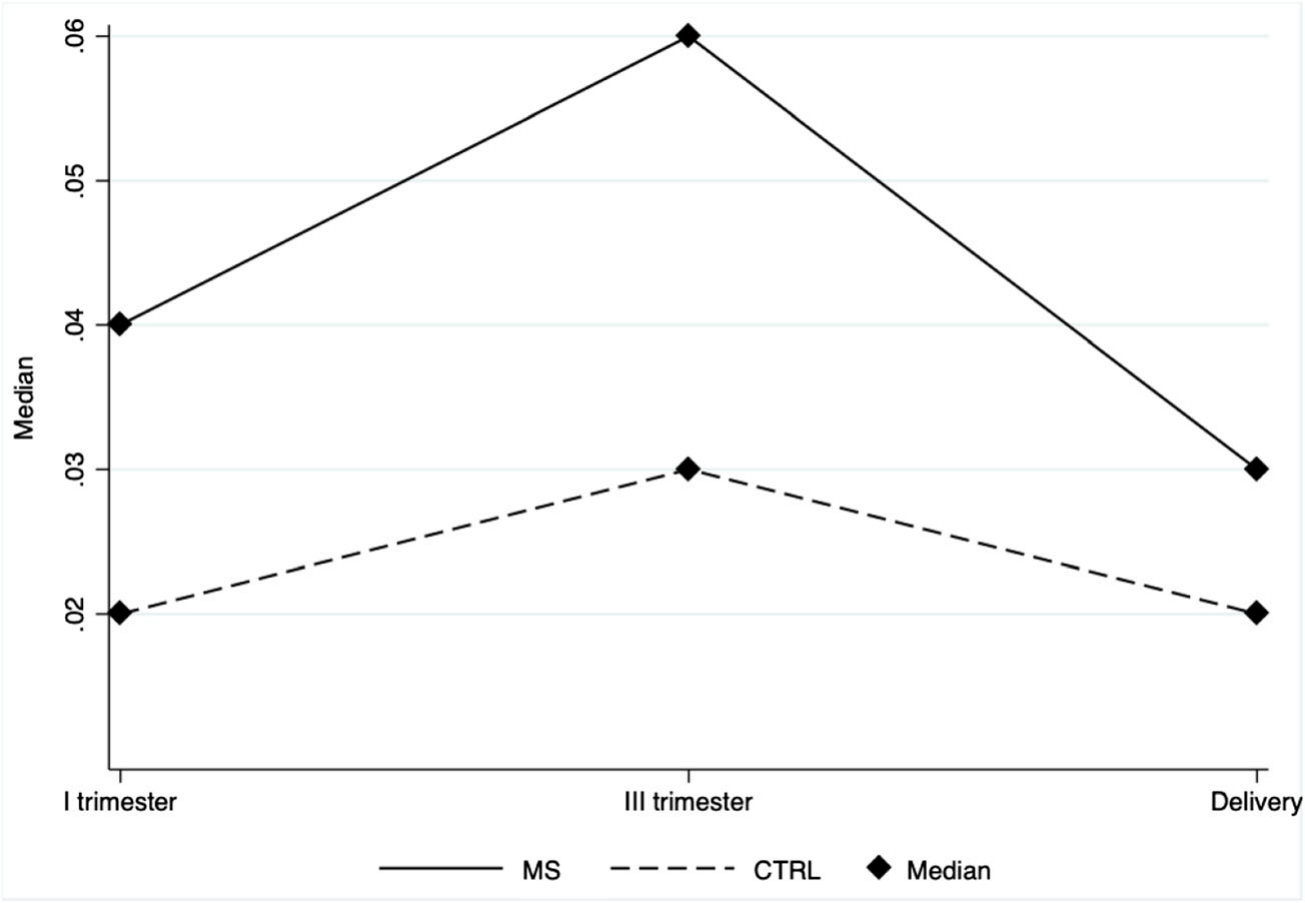
Profile plot depicting the median CD34+ cells in MS patients (solid line) and in healthy controls (CTRL) (dashed line) during pregnancy (x axis). The non-parametric analysis for repeated measures show that CD34+ cells levels during pregnancy are affected by MS (*p* = 0.0109) and by time (*p* = 0.0342). CTRL = healthy control; MS = multiple sclerosis

**Fig. 3:**
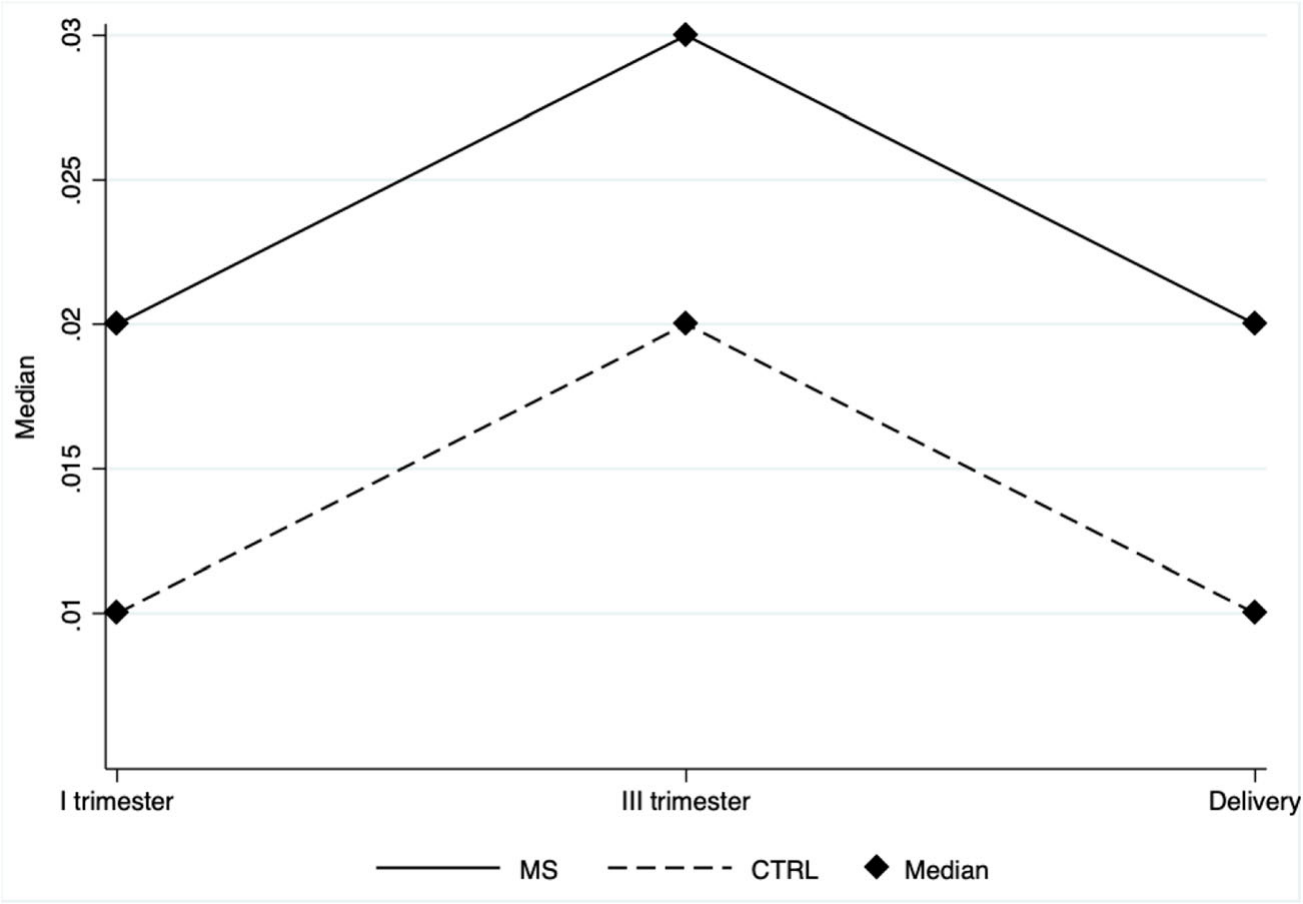
Profile plot depicting the median CD133+ cells in MS patients (solid line) and in healthy controls (CTRL) (dashed line) during pregnancy (x axis). The non-parametric analysis for repeated measures show that CD133+ cells levels during pregnancy are affected by MS (*p* = 0.0004) and by time (*p* = 0.0347). CTRL = healthy control; MS = multiple sclerosis

As illustrated in Fig. 2 and Fig. 3, both clinical status (MS versus healthy controls) and gestational age influenced progenitor cells levels during pregnancy.

EPCs were significantly higher in MS group compared with healthy controls. Specifically, the CD34+ median expression levels were 0.040 (IQR 0.030–0.046) among MS and 0.023 (IQR 0.020–0.026) among CTRL (*p* = 0.0004), whereas the CD133+ median expression levels were 0.233 (IQR 0.020–0.030) in the MS group and 0.016 (IQR 0.013–0.020) in the CTRL group (*p* = 0.0109).

In details, analysis highlighted that in MS group compared with healthy control group, mean CD34+ cells are increased by 47,9% (*p* = 0.030), 96.1% (p = 0.010), and 63.7% (*p* = 0.027) during the first trimester, the third trimester and at delivery, respectively (fig. 1 supplement). Mean CD133+ cells are increased by 50.3% (*p* = 0.190), 55.4% (*p* = 0.116) and 33.3% (*p* = 0.113) during the first trimester, the third trimester and at delivery, respectively (Fig. 2 supplement).

Additionally, time significantly affects levels of EPCs (CD34+: median first trimester 0.020 IQR 0.020–0.040, median third trimester 0.030 IQR 0.020–0.030; median at delivery 0.020 IQR 0.020–0.030, *p* = 0.0342; CD133+: median first trimester 0.015 IQR 0.010–0.020, median third trimester 0.020 IQR 0.010–0.030; median at delivery 0.010 IQR 0.010–0.020, p = 0. 0.0347).

In particular, analysis highlighted that CD34+ and CD133+ cells in CTRL group were lower at delivery compared with the third trimester (24.3% lower, *p* < 0.001 and 26.9%, *p* < 0.001, respectively); and CD133+ cells were higher during the third trimester compared with the first trimester (*p* = 0.047). No statistical difference was observed in MS group.

Progenitor levels at all time point were not associated with MS outcome (i.e. relapse free; number of relapses, radiological activity and EDSS) neither before pregnancy, nor during pregnancy, nor after pregnancy.

In both groups, we did not observe any correlation between progenitors and mean uterine artery Doppler PI, neither at first trimester nor at third trimester.

### Cord blood

A total of 38 observations (one observation per individual) were available for analysis. Clinical status (MS versus healthy controls) did not influence progenitors in cord blood.

Specifically, the CD34+ median expression levels were 0.390 (IQR 0.120–0.500) in MS group and 0.210 (IQR 0.170–0.240) in CTRL group (*p* = 0.203), whereas the CD133+ median expression levels were 0.240 (IQR 0.090–0.400) in the MS group and 0.120 (IQR 0.110–0.160) in the CTRL group.

**Fig. 4:**
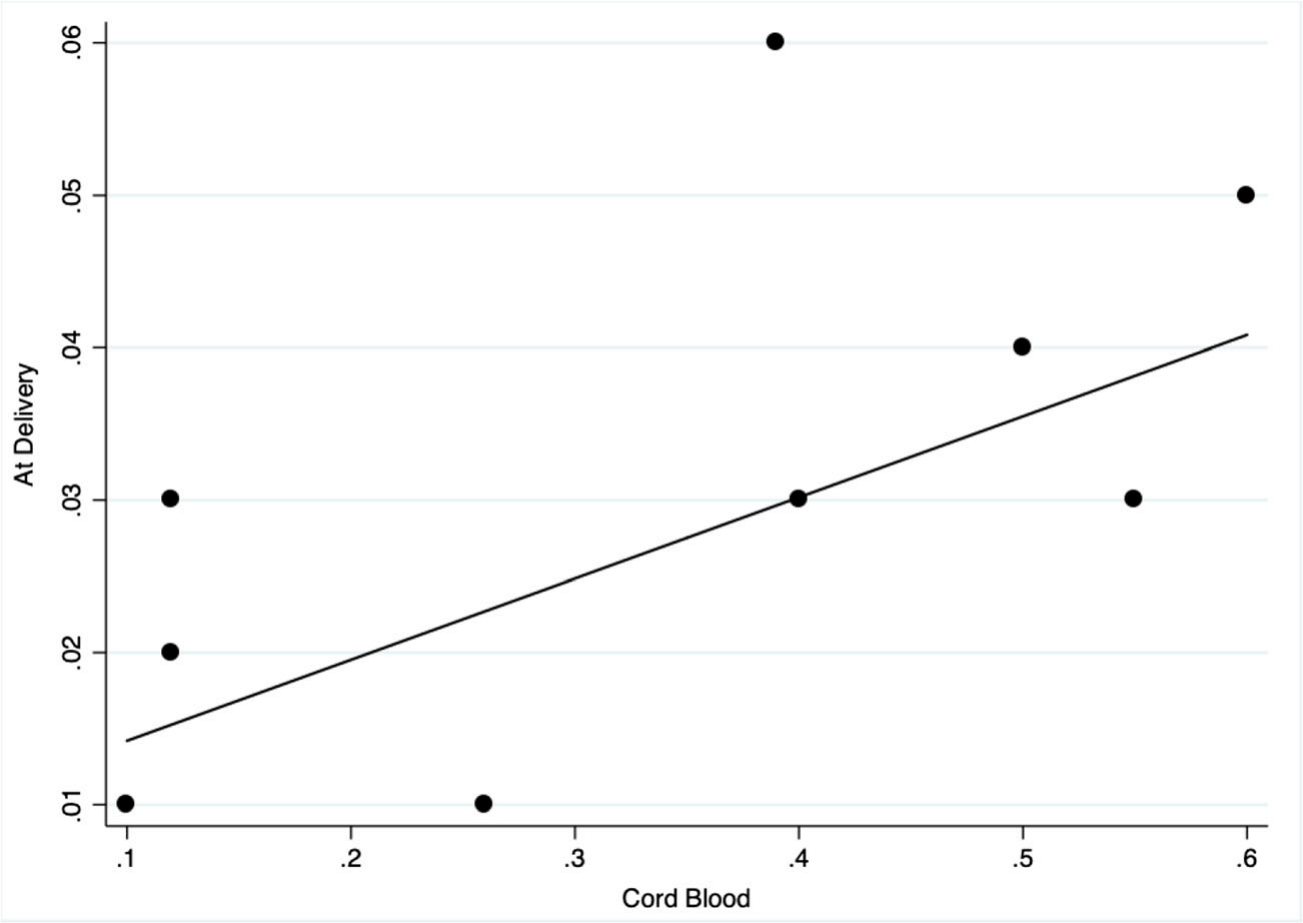
Spearman’s rank correlation scatter plot of CD34+ in the cord blood (x axis) and at delivery (y axis) in MS patients. The non-parametric analysis shows Rho 0.658, *p* = 0.054. MS = multiple sclerosis

**Fig. 5:**
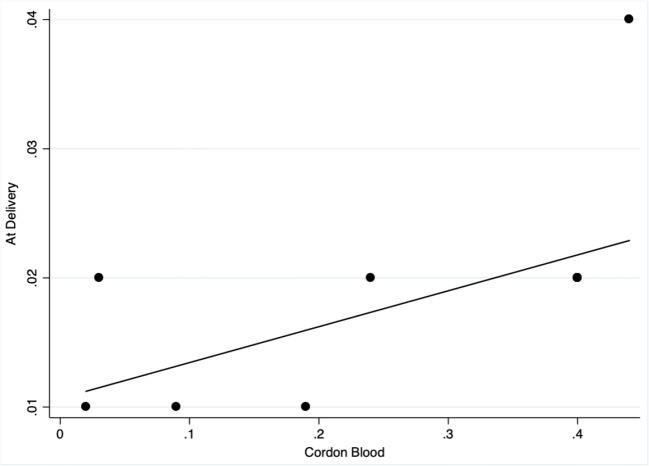
Spearman’s rank correlation scatter plot of CD133+ in the cord blood (x axis) and at delivery (y axis) in MS patients. The non-parametric analysis shows Rho 0.758, *p* = 0.018. MS = multiple sclerosis

Maternal CD34+ cells at delivery mild correlated with CD34+ in the cord blood neither in MS patients (spearman’s Rho 0.658, *p* = 0.054) but not in CTRL (spearman’s Rho 0.069, *p* = 0.723). Maternal CD133+ at delivery positively correlated with CD133+ in the cord blood in MS patients (spearman’s Rho 0.758, *p* = 0.018) but not in CTRL (spearman’s Rho 0.006, *p* = 0.976). Correlation plots are illustrated in Fig. 4 and Fig. 5.

## Discussion

Pregnancy in autoimmune diseases often requires a multidisciplinary approach to prevent and monitor maternal, foetal and neonatal issues [31, 32]. MS is no exception and pregnancy should be carefully planned, in order to optimize therapy and assess whether disease activity advises conception [33]. Contrary to several autoimmune diseases, MS patients are not at increased risk of pregnancy complications, such as preeclampsia, nor of general adverse obstetric outcome, nor of foetal or neonatal complications, even though some studies reported a slightly higher rate of preterm labour for these patients [6, 7]. In our study, there were no preterm labours. The evaluation of circulating EPCs may offer a hint on the marked difference in outcome favourability in MS patients with respect to patients affected by other autoimmune conditions.

Circulating EPCs are reduced in RA [16, 17], and the risk of spontaneous abortion seems to be higher in patients with RA [22] with respect to women without RA. In SSc, available data suggests that EPCs are recruited early in the course of disease during active inflammation, but as the disease progresses further, circulating EPC levels were shown to decrease, possibly as a result of their progressive depletion. [18–20]. Women with SSc present higher frequency of adverse events during pregnancy, including miscarriage, intrauterine growth restriction and gestational hypertension. [23]. Reduced levels of EPCs were found in patients affected by SLE [21]; in patients with SLE, pregnancy is considered at high risk, both for the maternal and foetal outcome [24].

Thus, in the present study, EPCs were evaluated during pregnancy in healthy women and patients with MS. To our knowledge, this is the first study which not only longitudinally analyses the expression of circulating EPCs in pregnant women with MS and in cord blood of their newborns but also which healthy controls and MS patients were referred to the same third level obstetric and third level MS Centre enhancing homogenous evaluation and data quality.

This work identified increased circulating EPC levels during pregnancy, following the same trend both in MS patients and in healthy controls. Despite the similar trend, the levels of circulating EPCs were significantly higher in MS patients with respect to the control population. Although the exact function of EPCs in pregnancy remains largely unexplored and requires further characterization, current knowledge suggests that these cells play a role in the correct development and functioning of the uteroplacental circulation promoting spiral artery remodelling. EPCs can be mobilized by several factors that notably increase during pregnancy, such as vascular endothelial growth factor, placental growth factor and oestrogen. To date, an increase in circulating EPCs during normal pregnancy has been reported by several studies [10–12]. Fewer authors observed an opposite trend, in contrast with the majority of literature [34].

The absence of a reduction in EPCs in MS pregnancies would appear to be in line with the non-increased risk of obstetric adverse events, as opposed to other conditions such as RA, SSc and SLE [6, 7]. Explaining the increased levels of EPCs observed in MS pregnant women is more challenging. Circulating EPC levels may be increased in MS patients, independently from pregnancy, or may be affected by a long-lasting effect of previous DMT [35]. Moreover, due to the small sample size of this study, results should be interpreted carefully.

Finally, to the best of our knowledge, there are no data about levels of circulating EPCs in the cord blood of newborns of MS patients. Cord blood EPCs can be affected by several maternal factors (such as gestational period, placenta weight, delivery way, ethnicity, foetal distress, maternal diseases) and neonatal factors (such as birth weight and sex) [36]. Pre-eclampsia seems to be associated with reduced levels of circulating EPCs in cord blood, while maternal hypertension has been sporadically associated with an increased number of circulating EPCs compared to healthy controls [37].

In this regard, this study found a mild correlation between circulating maternal CD34+ and CD133+ cells at delivery and circulating CD34+ and CD133+ cells in the cord blood of the newborns of MS patients. Although it is attempting to think that this result from a “carryover” effect, with passage from the maternal unit to the maternal-foetal unit, further studies are required to address this question.

Finally, although it is the object of research in multiple fields, EPC assessment has yet to be validated as a reliable marker of vascular dysfunction. However, it can be hypothesised that if EPCs will ever enter clinical practice as markers for the monitoring of pre-eclampsia or other pregnancy complications, the findings of this work may have relevant implications for this use of EPCs in women with MS. To this regard it is utmost importance to assess EPCs in larger groups of MS patients before conception and to evaluate EPC levels during their pregnancy and in post-partum. In fact, if women with MS tend to have higher circulating EPC levels during pregnancy with respect to healthy controls, the reference range or cut-off established in the general population may not be accurate for MS patients, who may be at increased risk of complications with higher EPCs levels, despite being in the normal range.

In conclusion, circulating EPC levels followed the same increasing trend in MS patients and healthy controls, with higher levels detected in MS patients. A correlation was found between cord blood EPCs and circulating EPCs at delivery. Further investigations in larger group of patients and focused on pre- and postpartum EPCs levels and on other inflammatory markers likely are required for the correct interpretation of these findings and to better examine the matter.

## Electronic supplementary material

ESM 1 (DOCX 110 kb)

## Data Availability

The data that support the findings of this study are available on request from the corresponding authors. The data are not publicly available due to privacy or ethical restrictions.
