# Supplementary material for: Circulating endothelial progenitor cells during pregnancy in multiple sclerosis
Source: Neurol Sci. 2020 Aug 17;42(4):1443–51. doi: 10.1007/s10072-020-04648-3 (PMC7956006; doi:10.1007/s10072-020-04648-3)
Supplement: Supplementary file 1 — (DOCX 110 kb) [file 10072_2020_4648_MOESM1_ESM.docx]

**Figure 1 supplement.** Mean ± SD CD34+ cell count/µL expressed as % in CTRL subjects (white) and in MS patients (black) in first trimester, in third trimester and at delivery. Mann-Whitney U test was performed to analysis the difference between CTRL and MS at each time point, p value reported

**Figure 2 supplement.** Mean ± SD CD133+ cell count/µL expressed as % in CTRL subjects (white) and in MS patients (black) in first trimester, in third trimester and at delivery. Mann-Whitney U test was performed to analysis the difference between CTRL and MS at each time point and p value reported if significant (p<0.05)
